# A Novel Method of Isolating Myofibrils From Primary Cardiomyocyte Culture Suitable for Myofibril Mechanical Study

**DOI:** 10.3389/fcvm.2019.00012

**Published:** 2019-02-19

**Authors:** Kathleen C. Woulfe, Claudia Ferrara, Jose Manuel Pioner, Jennifer H. Mahaffey, Raffaele Coppini, Beatrice Scellini, Cecilia Ferrantini, Nicoletta Piroddi, Chiari Tesi, Corrado Poggesi, Mark Jeong

**Affiliations:** ^1^Division of Cardiology, Department of Medicine, University of Colorado, Denver, CO, United States; ^2^Division of Physiology, Department of Experimental and Clinical Medicine, University of Florence, Florence, Italy

**Keywords:** myofibril, cell culture, mechanics, sarcomere, signaling

## Abstract

Myofibril based mechanical studies allow evaluation of sarcomeric protein function. We describe a novel method of obtaining myofibrils from primary cardiomyocyte culture. Adult rat ventricular myocytes (ARVMs) were obtained by enzymatic digestion and maintained in serum free condition. ARVMs were homogenized in relaxing solution (pCa 9.0) with 20% sucrose, and myofibril suspension was made. Myofibrils were Ca^2+^-activated and relaxed at 15°C. Results from ARVM myofibrils were compared to myofibrils obtained from ventricular tissue skinned with Triton X-100. At maximal Ca^2+^-activation (pCa 4.5) myofibril mechanical parameters from ARVMs were 6.8 ± 0.9 mN/mm^2^ (resting tension), 146.8 ± 13.8 mN/mm^2^ (maximal active tension, P_0_), 5.4 ± 0.22 s^−1^ (rate of force activation), 53.4 ± 4.4 ms (linear relaxation duration), 0.69 ± 0.36 s^−1^ (linear relaxation rate), and 10.8 ± 1.3 s^−1^ (exponential relaxation rate). Force-pCa curves were constructed from Triton skinned tissue, ARVM culture day 1, and ARVM culture day 3 myofibrils, and pCa_50_ were 5.79 ± 0.01, 5.69 ± 0.01, and 5.71 ± 0.01, respectively. Mechanical parameters from myofibrils isolated from ARVMs treated with phenylephrine were compared to myofibrils isolated from time-matched non-treated ARVMs. Phenylephrine treatment did not change the kinetics of activation or relaxation but decreased the pCa_50_ to 5.56 ± 0.03 (vehicle treated control: 5.67 ± 0.03). For determination of protein expression and post-translational modifications, myofibril slurry was re-suspended and resolved for immunoblotting and protein staining. Troponin I phosphorylation was significantly increased at serine 23/24 in phenylephrine treated group. Myofibrils obtained from ARVMs are a viable method to study myofibril mechanics. Phenylephrine treatment led to significant decrease in Ca^2+^-sensitivity that is due to increased phosphorylation of TnI at serine 23/24. This culture based approach to obtaining myofibrils will allow pharmacological and genetic manipulation of the cardiomyocytes to correlate biochemical and biophysical properties.

## Introduction

Since its development, myofibril mechanical assessment has led to advances in understanding of muscle function (1–4). Myofibril-based mechanical assays, compared to studies using larger preparations, offer several advantages. First, as the smallest ensemble of muscle motor proteins, myofibril mechanical experiments provide an evaluation of muscle mechanics without influence by non-motor protein systems such as those involved in calcium handling, energy metabolism, and the extracellular matrix. Second, the level of detail which myofibril mechanical experiments provide, such as resolution of the two phases of cardiac muscle fiber relaxation, is not possible with skinned cardiac fiber or intact cardiomyocyte preparations.

Traditionally, myofibrils are obtained from a small section of muscle that is either glycerinated or skinned in Triton X-100 and homogenized (5, 6). A major limitation of this approach is that these myofibrils are “as-is” with limited potential for biochemical manipulations. In this regard, specifically targeting a particular signaling pathway can only be achieved by generating transgenic animals, which is time-intensive and costly. The major strength of cardiomyocyte culture-based experiments is the ability to utilize readily available reagents and tools to address the myofilament mechanical consequences of altering a particular cellular function. Furthermore, with rapid advancement in stem cell-derived cellular systems, an *in vitro* method of obtaining myofibrils will provide a powerful experimental platform to better understand the pathobiology of diseases involving striated muscle. In this paper, we report a novel method of obtaining myofibrils from primary adult rat ventricular myocyte (ARVM) culture. We show that myofibrils obtained from primary ARVMs are equivalent to the traditional method and show applicability of this method to dissect the functional consequences of manipulating a specific signaling cascade.

## Methods

### Experimental Protocol

**Figure 1:**
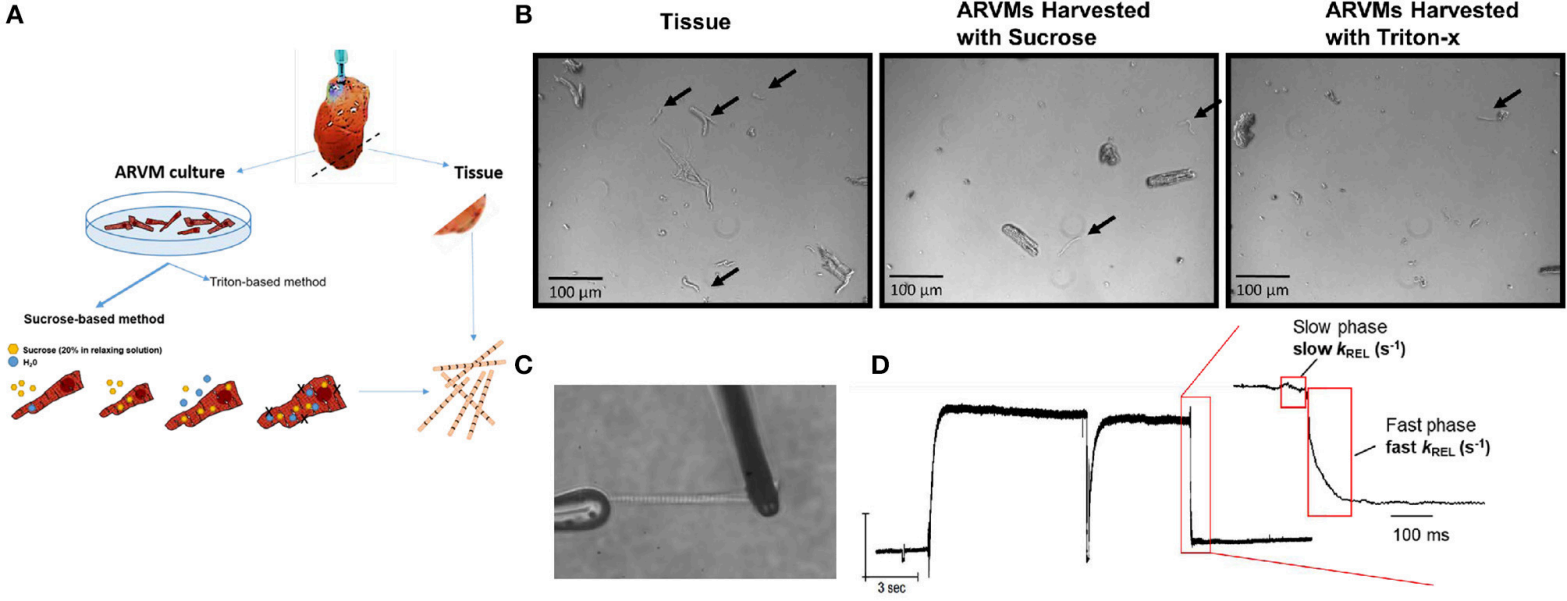
**(A)** Experimental scheme: hearts from Sprague Dawley rats are retrograde perfused. Before enzymatic digestion, a small section of the apex is cut to obtain myofibrils by skinning in Triton-X100. Remaining heart was digested in Liberase DH to obtained primary ARVM culture. Myofibrils from ARVM culture were obtained by sucrose based osmotic shock method. **(B)** Representative images of 10x magnification demonstrating the different quantity and quality of myofibrils isolated using the different techniques. Black arrows indicate useable myofibrils in each field. The myocyte fragments shown in the panel of ARVMs harvested using Triton-x show a morphology distinct from the fragments of the other harvest techniques. **(C)** Representative image of a myofibril isolated from ARVMs using the sucrose-based method. **(D)** Representative trace from an ARMV-derived myofibril activated and relaxed by fast solution switching. ARVM, adult rat ventricular myocyte.

Adult rat left ventricular myocytes (ARVMs) were obtained from female Sprague Dawley rats (250–300 g) (7). Animal studies were reviewed and approved by University of Florence and University of Colorado Institutional Animal Care and Use Committee (IACUC) thereby meeting the standards set by the Directive 2010/63/EU of the European Parliament on the protection of animals used for scientific purposes and the NIH standards for the care and use of laboratory animals. The heart was rapidly removed and retrograde perfused with perfusion buffer (120.5 mM NaCl, 14.7 mM KCl, 0.6 mM KH_2_PO_4_, 0.6 mM Na_2_HPO_4_, 1.2 mM MgSO_4_, 4.6 mM NaHCO_3_, 10 mM Na-HEPES, 30 mM Taurine, 10 mM 2,3-butanedione monoxime, 5.5 mM Glucose, pH 7.2) for 10 min at 37°C. A small section of the left ventricular apex was cut at the end of the pre-digestion perfusion. The small apical tissue was skinned in Triton X-100. The remainder of the heart was enzymatically digested to make ARVMs (Figure 1).

### Cardiomyocyte Culture

The heart was digested with Liberase DH (Roche, 0.33 mg/ml) for 8 min, cut into small pieces, and a slurry was filtered through sterile 150-nm mesh. The filtrate was centrifuged at 400 g for 4 min to separate myocytes from non-myocytes. The myocyte suspension was layered over 60 μg/ml of BSA and allowed to settle for 15 min to separate myocytes from non-myocytes. Myocyte concentration was determined and plated on 100 mm laminin-coated plastic culture dishes at a density of 100 to 150 cells/mm^2^. The ARVM culture was maintained in serum-free DMEM supplemented with albumin (2 mg/ml), carnitine (2 mmol/l), creatine (5 mmol/l), taurine (5 mmol/l), BDM (1 mg/ml), and penicillin-streptomycin (100 μg/ml).

### Myofibrils From Cardiomyocyte Culture

ARVMs were washed in sterile PBS (room temperature) twice then collected one of two ways. (1) The cells were collected in 1 ml of cold 20% sucrose in relaxing solution (pCa 9.0) with protease inhibitor cocktail (10 μM leupeptin, 5 μM pepstatin, 200 μM phenyl-methylsuphonylfluoride, 10 μM E64, 500 μM NaN3, 2 mM dithioerythritol). The demembraned ARVM slurry was vortexed (30 s then settled on ice for 10 min. The cell suspension was centrifuged (300 g for 15 min in 4°C) and the pellet was resuspended in fresh relaxing solution with protease inhibitor cocktail to remove the sucrose. The resuspension/wash cycle was repeated twice. The final demembraned ARVMs were resuspended in relaxing solution with protease inhibitor cocktail and homogenized at medium speed (Tissue Tearer) for 15–20 s to make the final myofibril suspension. (2). Cells were collected in 1 ml of cold 0.01% Triton-X in relaxing solution (pCa9.0) with protease inhibitor cocktail. The ARVM slurry was treated as described above.

### Myofibrils From Apical Tissue

The left ventricular apex was cut into thin slices and bathed in 0.05% Triton X-100 and protease inhibitor cocktail in rigor solution overnight at 4°C. The skinned tissue was then washed twice in rigor solution with protease inhibitor cocktail with gentle shaking. The skinned tissue was resuspended in 1 ml rigor solution with protease inhibitor cocktail and homogenized at medium speed for 15–20 s to make the final myofibril suspension (8, 9).

### Myofibril Mechanical Study

We used previously published techniques to measure and control the force and length of isolated myofibrils activated and relaxed by fast solution switching (8, 9). Briefly, a small volume of myofibril suspension was transferred to a temperature controlled chamber (15°C) filled with relaxing solution (pCa 9.0). A small bundle of myofibrils were mounted between two microtools. One tool was connected to a motor that could produce rapid length changes (Mad City Labs). The second tool was a calibrated cantilevered force probe (4–8 μm/μN; frequency response 2–5 KHz). Myofibrils were set 5–10% above slack myofibril length in relaxed conditions (pCa 9). Average sarcomere length and myofibril diameter were measured using ImageJ. Mounted myofibrils were activated and relaxed by rapidly translating the interface between two flowing streams of solutions of different pCa (9, 10). Data was collected and analyzed using a customized LabView software. Measured mechanical and kinetic parameters were defined as follows: resting tension (mN/mm^2^)—myofibril basal tension in fully relaxing condition (pCa 9.0); maximal tension (mN/mm^2^)—maximal tension generated at full calcium activation (pCa 4.5); the rate constant of tension development following maximal calcium activation (*k*_ACT_); the rate constant of tension redevelopment following a release-restretch applied to the activated myofibril (*k*_TR_) (11); rate constant of early slow force decline (slow *k*_REL_)—the slope of the linear regression normalized to the amplitude of relaxation transient, duration of early slow force decline—measured from onset of solution change to the beginning of the exponential force decay, the rate constant of the final exponential phase of force decline (fast *k*_REL_). To determine force-pCa relationship, peak isometric force was recorded at maximal (pCa 4.5) and submaximal (pCa 5.2, 5.4, 5.6, 5.8, 7) calcium concentrations and fitted to the Hill equation (P/P0 = 1/(1 +10^(−nh(pCa50−pCa))^).

### Myofilament Protein Analysis

To determine the level of phosphorylated myofilament proteins, the myofibril slurry was spun down and protein pellet was resuspended in isoelectric focusing buffer (IEF; 8 M Urea, 2.5 M Thiourea, 4% Chaps, 2 mM EDTA, 1 mM DTT, 1% TBP, phosphatase, and protease inhibitors). Twenty micrograms of protein was resolved by SDS-PAGE using a 10% gel and stained with ProQ Diamond. The gel was washed in methanol and restained with commassie brilliant blue (CBB) to determine total protein abundance. Gels were scanned and densitometry was measured using ImageJ. To determine the site-specific phosphorylation of troponin I, Western blots were performed. Site specific phospho-antibodies directed to serine 23/24 (Phosphosolutions p2010–2324, 1:1,000) and serine 43/44 (Phosphosolutions 9056, 1:1,000) of troponin I were used for Western blotting. TnI antibody (Fitzgerald 10R-T123K, 1:1,000) was used to determine the relative abundance of phosphorylated proteins. Densitometry data was used to normalize the phospho-specific signal over the total TnI signal.

### Statistical Analysis

Statistical analyses were performed using Prism Version 6 (GraphPad Software, San Diego, CA). Mean ± SEM values are presented. Data were checked for normality by Shapiro-Wilks test and if normal, compared by Student *t*-test (2 unpaired groups) or one-way ANOVA (>2 groups) with Newman-Keuls post-test. If not normal, data were log-transformed before statistical testing. Probability values of *p* < 0.05 were considered significant.

## Results

### Myofibrils From Cultured Cardiomyocytes Permeabilized Using Sucrose Are Viable

Our initial aim was to determine the cardiomyocyte skinning buffer and homogenization protocol to harvest myofibrils from ARVMs. We began with low concentrations of Triton X-100 in rigor or relaxing solutions. 0.1% Triton X-100 caused complete lysis of cardiomyocytes in either buffer. We were able to harvest myofibrils from cardiomyocytes skinned in 0.01% Triton X-100 in rigor solutions but yielded poor quality and lower quantity of myofibrils. These myofibrils tended to be shorter, more fragmented, and difficult to mount on our force recording machine (Figure 1B). Sucrose (20% by volume) in relaxing solution was able to disrupt the cellular membrane without affecting the integrity of the myofibrils (Figures 1B–D).

### Mechanical Parameters From Myofibrils From ARVM Are Comparable to the Mechanical Parameters From Myofibrils From Tissue

**Figure 2:**
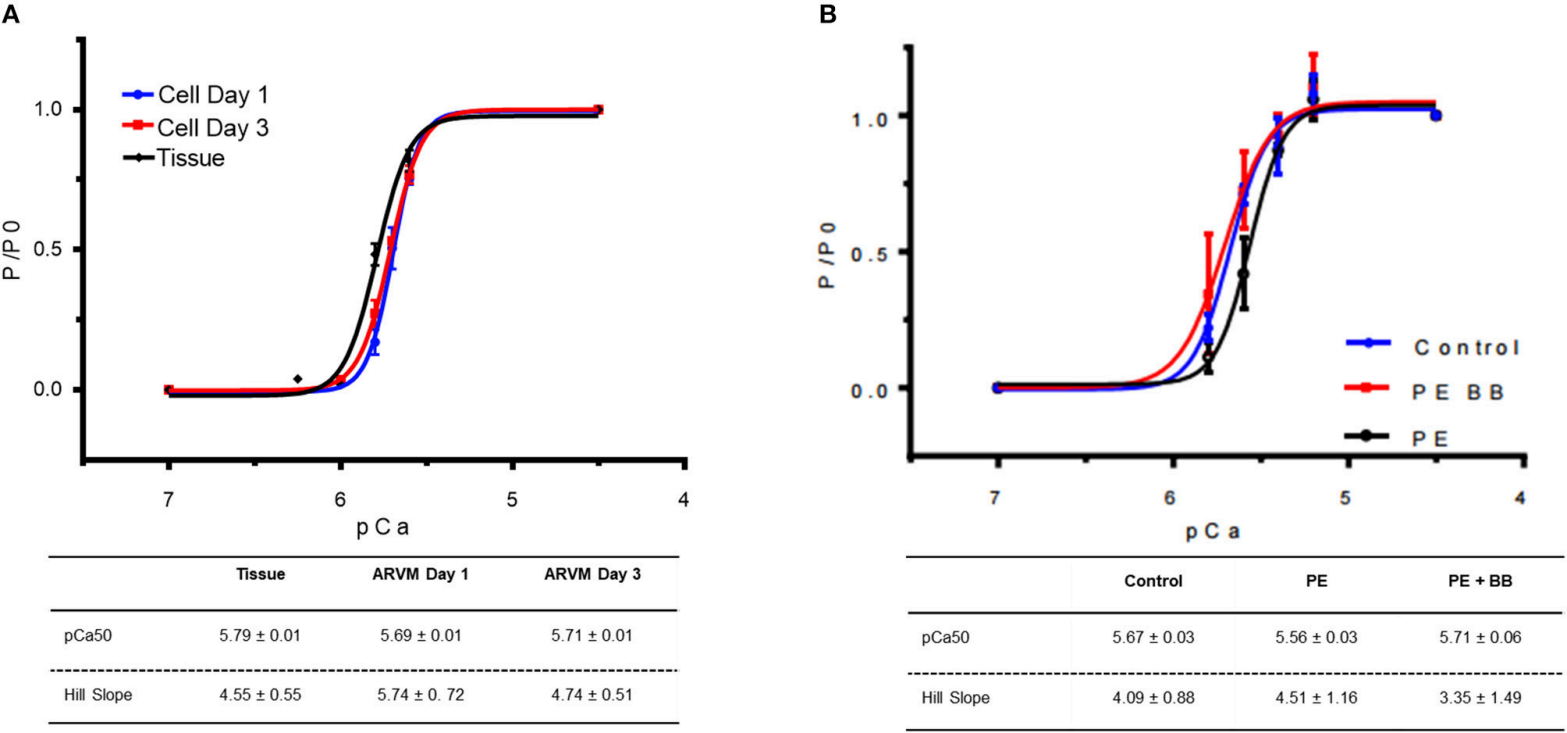
**(A)** Force-pCa relationship of myofibrils obtained from cardiomyocyte culture day 1 (blue), culture day 3 (red) vs. myofibrils isolated from cardiac tissue (black). At each pCa, maximal tension was normalized to tension generated at pCa 4.5 (P_0_). Data points are mean ± SEM. Lines are drawn according to the parameters estimated by fitting to the Hill equation (P/ P_0_ = 1/)(1 +10^(−nh(pCa50−pCa))^). N = 4 rats, 16–20 myofibrils. **(B)** Force-pCa relationship of myofibrils obtained from ARVMs that were PE-treated (black), myofibrils treated with PE and the β-blocker propranolol (BB) (red) vs. vehicle treated control (blue). *N* = 6 rats, 16–22 myofibrils. ARVM, adult rat ventricular myocyte; PE, phenylephrine; BB, β-blocker propranolol.

Next mechanical parameters of myofibrils from ARVM culture harvested with sucrose or frozen tissue were compared. Myofibrils were isolated from frozen rat LV as a comparison since this is the standard source of myofibrils used to assess disease-specific differences in human hearts or animal models. The isometric sarcomere length measured was similar. The mechanical and kinetic behavior of ARVM myofibrils was not statistically different from tissue-derived myofibrils for most parameters (Table 1). The kinetics of force redevelopment (*k*_TR_) were, however, slower in myofibrils from tissue. Mechanical run-down, the decrement of force generation, and its kinetics with successive activation-relaxation cycles was comparable between myofibrils isolated from ARVMs and tissue (data not shown). Force-pCa curves were constructed from myofibrils obtained from ARVMs in culture or from tissue. Ca^2+^-sensitivity and the Hill coefficients (nH) were similar between myofibrils from ARVM and tissue (Figure 2A).

**Table 1:** Comparison of mechanical parameters between myofibrils obtained from ARVM culture vs. traditional small tissue section.

	**ARVM (N, *n*)**	**Tissue (N, *n*)**	***P***
Sarcomere length (μm)	2.24 ± 0.09 (6, 19)	2.28 ± 0.07 (6, 15)	ns
Resting tension (mN/mm^2^)	6.8 ± 3.5 (6, 17)	6.6 ± 3.8 (6, 13)	ns
Maximal tension (mN /mm^2^)	146.8 ± 51.7 (6, 14)	139.6 ± 51.3 (6, 15)	ns
*K*_ACT_ (s^−1^)	5.37 ± 0.95 (6, 18)	4.97 ± 1.14 (6, 14)	ns
*K*_TR_ (s^−1^)	5.20 ± 0.89 (6, 17)	4.52 ± 0.82 (6, 15)	*p* < 0.05
Linear relaxation duration (ms)	53.4 ± 17.7 (6, 16)	52.0 ± 16.2 (6, 12)	ns
Exponential relaxation rate (s^−1^)	10.8 ± 4.8 (6, 14)	14.6 ± 6.5 (6, 12)	ns

*Myofibrils are fully calcium activated (pCa 4.5) and fully relaxed (pCa 9) by fast solution switching. Data presented with mean ± SEM. (N,n), (N# of rats, n # of myofibrils)*.

### Duration of Cultured Cells Affects Kinetics of Activation and Relaxation but Not Calcium Sensitivity

**Figure 3:**
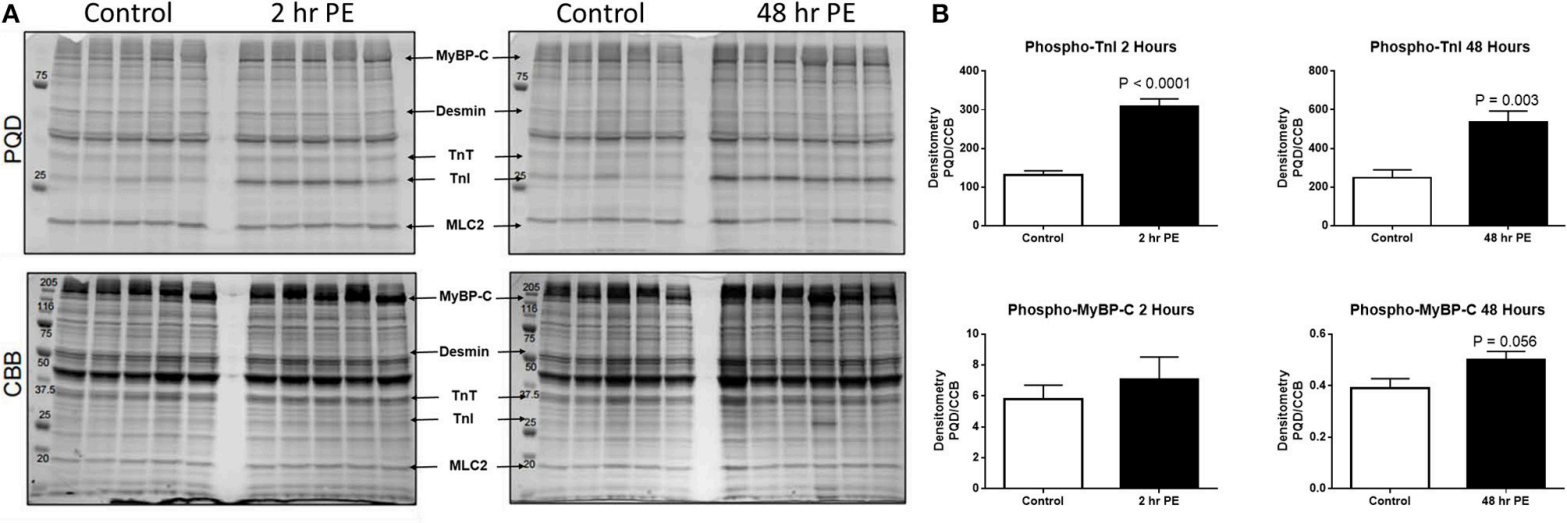
**(A)** Myofilament protein phosphorylation from ARVMs treated with PE for 2 and 48 h. ProQ Diamond® stain (PQD) was used to determine the global phosphorylation status of myofilament proteins. The same gel was stained with commassie brilliant blue (CBB) to determine relative abundance of proteins. **(B)** Quantification of PQD over CBB densitometries. Only those sarcomeric proteins with significant change in global phosphorylation are shown. Phosphorylation of TnI is significantly increased in ARVMs after 2 h (*p* < 0.0001) and 48 h (*p* = 0.003) of PE treatment. Myosin binding protein C (MyBP-C) trends to increased phosphorylation at 48 h of treatment (*p* = 0.056) but not at 2 h. *N* = 5 rats. ARVM, adult rat ventricular myocyte; PE, phenylephrine; CBB, commassie brilliant blue; TnI, troponin I; MyBP-C, myosin binding protein C.

In order to understand how prolonged culture affects the mechanical properties of cardiac myofibrils, we compared myofibrils isolated using sucrose from cardiomyocyte culture at day 1 and day 3. Myofibrils from day 1 group were isolated from cardiomyocytes that were kept in serum-free media in a 37°C incubator between 2 and 8 h. The 3-day-old cardiomyocytes were kept in a serum-free culture environment for an additional 48 h. Although magnitude of tension generation was unaffected by long-term culture, 3 days of culture showed changes in kinetics of activation and relaxation (Table 2). Using Pro-Q Diamond staining to assess for phosphorylation of sarcomeric proteins, we did not see a significant change in the degree of phosphorylation of key contractile and regulatory proteins of the sarcomere (Figure 3). The duration that ARVMs were kept in primary culture had no impact on Ca^2+^-sensitivity or the Hill coefficient (Figure 2A).

**Table 2:** Comparison of mechanical parameters between myofibrils obtained from ARVMs cultured for 1 day and ARVMs maintained in serum-free medium for 3 days.

	**ARVM Day 1 (N, *n*)**	**ARVM Day 3 (N, *n*)**	***P***
Sarcomere length (μm)	2.24 ± 0.09 (6, 19)	2.25 ± 0.07 (8, 9)	ns
Resting tension (mN/mm^2^)	6.8 ± 3.5 (6, 17)	7.3 ± 3.2 (8, 19)	ns
Maximal tension (mN /mm^2^)	146.8 ± 51.7 (6, 14)	135.8 ± 47.6 (8, 18)	ns
*K*_ACT_ (s^−1^)	5.37 ± 0.95 (6, 18)	4.05 ± 0.98 (8, 19)	*P* < 0.05
*K*_TR_ (s^−1^)	5.20 ± 0.89 (6, 17)	4.90 ± 0.95 (8, 19)	ns
Linear relaxation duration (ms)	53.4 ± 17.7 (6, 16)	34.2 ± 8.5 (8, 14)	*P* < 0.05
Exponential relaxation rate (s^−1^)	10.8 ± 4.8 (6, 14)	10.2 ± 5.2 (8, 16)	ns

*Myofibrils are fully calcium activated (pCa 4.5) and fully relaxed (pCa 9) by fast solution switching. Data presented with mean ± SEM*.

### Treating ARVMs With Phenylephrine Leads to Decreased Calcium Sensitivity in Myofibrils Due to Phosphorylation of Troponin I

**Figure 4:**
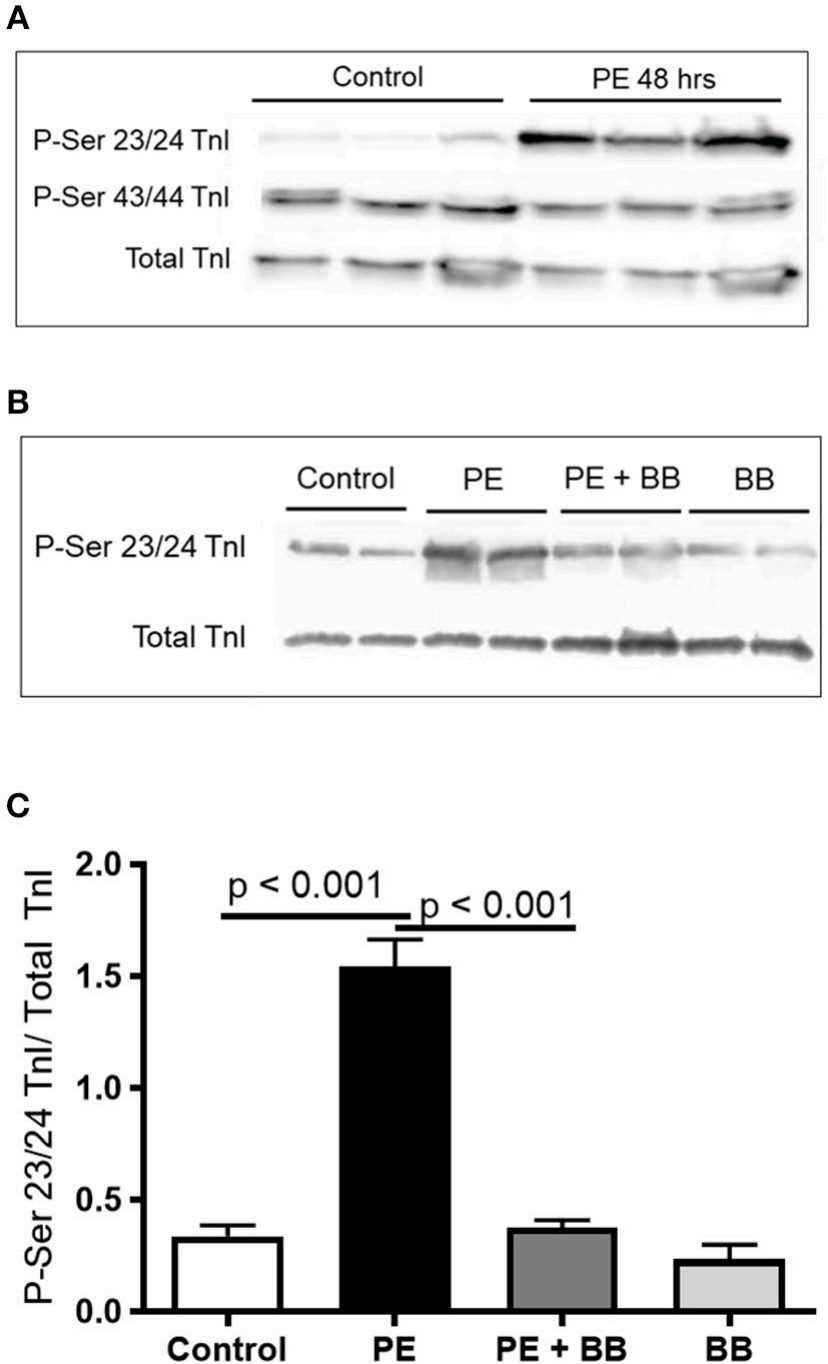
**(A)** Representative Western blot demonstrating serine 23/24 is preferentially phosphorylated with PE treatment. Each lane is protein isolated from different experiments. These conditions were repeated in ARVMs cultured from 5 different rat hearts. **(B)** Representative image of a Western blot showing pre-treatment of ARVMs with the β-blocker propranolol (BB) leadsto inhibition of TnI phosphorylation at serine 23/24. Experiment was repeated in ARVMs cultured from 3 different rat hearts. **(C)** Quantification of TnI serine 23/24 phosphorylation normalized to total TnI. PE significantly increases phosphorylation of TnI at Ser 23/24 (*p* < 0.001). Treatment with PE and a β-blocker (BB) propranolol, normalizes TnI phosphorylation (PE tx v PE+BB *p* < 0.001). Each lane is protein isolated from different experiments. These conditions were repeated in ARVMs cultured from 3 different rat hearts. TnI, troponin I; PE, phenylephrine; ARVM, adult rat ventricular myocyte; BB, β-blocker propranolol.

To demonstrate the applicability of culture based cardiomyocyte myofibrils to study the effect of manipulating the adrenergic signaling pathway, we treated ARVMs with phenylephrine (PE; 20 μM) for 48 h and performed myofibril mechanical measurements and sarcomeric protein analysis. Myofibrils were harvested using the sucrose method. We and others have shown that prolonged PE treatment leads to significant cardiomyocyte hypertrophy, induction of the pathologic gene profile, and deterioration of intact cardiomyocyte function (12, 13). The kinetics of activation and relaxation, and tension generation were unchanged with PE treatment (Table 3). Treatment with PE led to a significant decrease in calcium sensitivity (Figure 2B). Using CBB protein staining, we did not see a change in the expression level of sarcomeric proteins between vehicle vs. PE treated ARVMs (Figure 3). Global phosphorylation status was examined using Pro-Q Diamond stain. PE treatment led to increased phosphorylation of troponin I (TnI) (Figure 3). Western blot analysis confirmed the change in phosphorylation of serine 23/24 while serine 44 was not affected (Figure 4A). Decreased calcium sensitivity due to phosphorylation of TnI at serine 23/24 is consistent with previously published studies. To confirm our hypothesis that PE was also activating PKA via β-adrenergic signaling pathway, we treated ARVMs with PE with or without propranolol (2 μM). Propranolol treatment was sufficient to inhibit the increased phosphorylation of Serine 23/24 TnI (Figure 4) and the shift in pCa50 (Figure 2B). Propranolol treatment alone did not have any effect in basal expression of TnI or phosphorylation (Figure 4).

**Table 3:** Comparison of mechanical parameters between myofibrils from ARVMs treated with PE for 48 h.

	**Vehicle (N, *n*)**	**PE (N, *n*)**	***P***
Sarcomere length (μm)	2.21 ± 0.1 (6, 19)	2.21 ± 0.1 (6, 22)	ns
Resting tension (mN /mm^2^)	7.6 ± 2.8 (6, 18)	6.7 ± 2.5 (6, 17)	ns
Maximal Tension (mN /mm^2^)	135.8 ± 47.6 (6, 17)	118.8 ± 38.6 (6, 17)	ns
*K*_ACT_ (s^−1^)	4.05 ± 0.98 (6, 19)	3.95 ± 0.94 (6, 19)	ns
*K*_TR_ (s^−1^)	4.90 ± 0.95 (6, 19)	4.37 ± 1.02 (6, 19)	*P* = 0.059
Linear Relaxation Duration (ms)	34.2 ± 8.5 (6, 15)	43.8 ± 17.1 (6, 15)	ns
Linear Relaxation Rate (s^−1^)	1.27 ± 0.91 (6, 6)	1.35 ± 0.65 (6, 8)	ns
Exponential Relaxation Rate (s^−1^)	9.04 ± 3.3 (6, 15)	10.0 ± 5.1 (6, 19)	ns

*Myofibrils are fully calcium activated (pCa 4.5) and fully relaxed (pCa 9) by fast solution switching. Data presented with mean ± SEM. (N,n), (N# of rats, n # of myofibrils)*.

## Discussion

We report a novel method of obtaining myofibrils from cultured ARVMs which are suitable for myofibril mechanical experiments. We establish that myofibrils obtained from an ARVM culture system are equivalent in mechanical properties to myofibrils prepared by skinning small cardiac tissue with Triton-X100. Demonstrating applicability of this method, we show that PE treatment does not significantly affect activation kinetics and tension generation, but PE treatment increases phosphorylation of TnI contributing to decreased myofilament calcium sensitivity when compared to vehicle treated controls. These findings show that primary ARVM culture system can be used for myofibril mechanical studies, and will expand the utility of myofibril mechanical experiment by providing a wider palette of experimental conditions.

The major advantage of myofibrils obtained from a primary culture system over the traditional Triton X-100 method is that primary culture system allows manipulation of specific cellular processes using widely available experimental techniques. The traditional method of obtaining myofibrils from a small heart section can be viewed as “as-is” with limited potential for *ex-vivo* manipulation. The combination of myofibril mechanical experiments and the wide array of biochemical techniques available to manipulate cultured cells provides a flexible experimental system to study the mechanical consequences of altering a specific cellular function. ARVM based myofibril studies provides additional advantages. ARVM are relatively inexpensive when compared to the need for generating mutant animals. For example, if you consider a potential experiment studying how protein x impacts myofibril mechanics, the cost and time to generate a transgenic or knockout animal is roughly $7,000 and can take up to 14 weeks. From there, there are costs associated with breeding and maintenance of a colony where animal costs can exceed $250 a month not including the salary of the staff necessary to oversee the colony. This total quickly reaches $10,000 or more and takes at least 6 months. In contrast, consider purchasing 6 adult rats at roughly $50 each and generating ARVMs. From here, the cells can be treated with inhibitors or ectopically expressed proteins and myofibril mechanics studied. On a practical level, we found that homogenizing intact cardiomyocytes to small bundles of myofibrils was easier to achieve from ARVMs than from small cardiac tissue. Our ARVM based method is complementary to the current standard and provides an additional source of experimental material. Importantly, our results show that myofibrils from ARVMs are mechanically comparable to myofibrils derived from tissue.

One difference in mechanical properties that we noted between tissue and ARVMs harvested using sucrose was that k_TR_ was faster in ARVMs. This difference could be due to structural changes in the sarcomeres that occur following plating (14, 15) or it may be an experimental difference in isolation techniques (Triton X-100 compared to sucrose). It is important to recognize that these factors affect the mechanical parameters and experimental conditions should be considered appropriately. To further explore how plating impacts sarcomere function, we determined how duration of primary culture impacts myofibril function. Cardiomyocytes have been found to undergo transformation when maintained in primary culture. Although maintaining cardiomyocytes for up to 15 days has been described (15), we and other investigators report an optimal culture time frame of up to 5–8 days (7). It is believed that the limited life-span of ARVMs in culture is due to dedifferentiation. Eppenberger et al. (16) demonstrated that over time cultured ARVMs have extensive morphological changes so that they look more like fetal myofibril and therefore achieve a less differentiated state (16). Dedifferentiation of cardiomyocytes in long-term culture is inevitable, but serum-free culture media decreases the rate of dedifferentiation (14). The culture media is critical to maintain healthy cells, and the culture media additives for ARVMs are albumin, creatine, carnitine, taurine, BDM, penicillin, and streptomycin. We have set 5 days as the upper limit for maintaining ARVMs in culture, and have designed our experiments around this time frame. We and others have previously shown and show additional data in this report that key functional and regulatory proteins of the sarcomere are unchanged in the level of expression and modification (12, 13, 17). The half-life of sarcomeric proteins ranges between 3 and 10 days (18, 19). Our data shows that there is no significant change in the expression level or global phosphorylation of sarcomeric proteins obtained from ARVMs in short vs. long term culture. Although we do not report qualitative or quantitative changes of sarcomeric proteins, we report significant changes in activation and relaxation kinetics between day 1 and day 3. This degree of change in biomechanical properties is not unexpected considering that ARVMs in primary culture are dedifferentiating. We hypothesize that routine biochemical assays used in this study to evaluate for the abundance and the post-translational modification status of sarcomeric proteins are not sensitive enough to detect subtle changes. These small changes appear to be sufficient to exert significant change in myofilament function. In this regard, experiments that compare myofibril mechanics between freshly isolated ARVM to one that is several days old would be inappropriate. In our experimental protocol, treated groups are always compared to age-matched vehicle treated controls, thereby allowing us to evaluate the effects of the treatment group while taking into account the unavoidable changes that are inherent in a primary culture based experiment.

Adult rat ventricular cardiomyocytes treated with PE (20 μM) for 48 h demonstrate that TnI phosphorylation is an important effector of sarcomeric protein function. Previously, we and others have shown that treating ARVMs with 48 h of PE caused contractile dysfunction (7, 13). This report builds on the previous study and provides new data showing that cardiomyocyte dysfunction does not require dysfunction of myofibril contractile machinery. This observation suggests a hierarchy of cardiomyocyte dysfunction by showing that the contractile machinery remains capable even in the face of cellular dysfunction. If the basic contractile unit from a dysfunctional cardiomyocyte is functioning normally, then can we target the basic contractile unit to normalize the whole cell function? We have previously published data showing that targeted inhibition of histone deacetylase 6 can augment contractility of myofibrils to normalize whole heart function in an angiotensin model of rodent heart failure (20). This strategy of targeting the myofibril to augment inotrope may offer benefits over currently available agents such as dobutamine or milrinone which targets the upstream receptor or enzymatic cascade immediately below the receptor. Clinical data may support this hypothesis since in adult heart failure literature, prolonged use of dobutamine or milrinone, while having positive inotropic effects, leads to increased mortality from side effects (21, 22). If our observation can be applied to human heart failure, perhaps targeting the myofibrillar proteins directly may provide a more direct and less toxic inotropic support.

We also report that PE, an α-adrengeric and β-adrenergic agonist (23), modifies TnI at serine 23/24. TnI serine 23/24 is predominantly under the control of protein kinase A (PKA), the down-stream effector of β-adrenergic receptor cascade. This observation provides insight into the role of PKA in modulating troponin I phosphorylation, the kinetics of cross-bridge cycle, and calcium sensitivity of cardiac myofibrils. We show that increased phosphorylation of serine 23/24 TnI decreases the calcium sensitivity without affecting the kinetics of activation or relaxation. Our results are in agreement with previous work performed in human myofibrils treated with PKA (24). Others, using rodent myofibrils, showed that activation of PKA resulted in both right shift in calcium sensitivity and changes in activation and relaxation kinetics (25, 26). Specifically, Rao et al. (25) exchanged TnI with targeted mutations in serine 23/24 into myofibrils to illustrate that phosphorylation status of TnI can control both relaxation duration and calcium sensitivity of activation. While both studies show the regulatory role of serine 23/24 in calcium sensitivity of activation, PE treatment of ARVMs leads to different results in relaxation kinetics. This difference may be due to the experimental condition. In our experiment, we stimulated cardiomyocytes with an upstream agonist thereby activating myriad of other secondary effectors. In Rao's work, exchanging mutant TnI into myofibrils would avoid the effects of signaling cascade that results from receptor activation (25). Regardless of the experimental protocol, we are in agreement that serine 23/24 is an important effector in changing sensitivity to calcium activation. Additional conclusions about the role of serine 23/24 in regulating relaxation remains, however, unsettled. The ability to obtain viable myofibrils from ARVMs presents an opportunity to use established myofibril mechanical assays in an *in vitro* model system which will be a valuable tool to inform mechanisms driving mechanical alterations.

In conclusion, we report that myofibrils obtained from ARVM culture are similar in mechanical properties to myofibrils isolated by skinningsmall sections of heart tissue. While the traditional method of Triton X-100 based myofibrils will remain germane to myofibril mechanical research, we are confident that the primary ARVM culture based myofibrils will prove to be a powerful research tool complementing the time proven traditional method.

## Author Contributions

MJ, CFa, JM, BS, KW, and NP performed myofibril mechanical experiments. MJ, JP, RC, and CFi isolated adult rat ventricular myocytes and myofibrils. MJ and CP designed and built the photodiode motion detector. MJ, CFi, and RC wrote the data acquisition program. MJ and JM performed protein analysis. MJ, KW, CT, and CP designed the experiments and analyzed all data. All authors contributed to writing the manuscript.

### Conflict of Interest Statement

The authors declare that the research was conducted in the absence of any commercial or financial relationships that could be construed as a potential conflict of interest.
